# Identification and *in vitro* characterization of two new PCSK9 Gain of Function variants found in patients with Familial Hypercholesterolemia

**DOI:** 10.1038/s41598-017-15543-x

**Published:** 2017-11-10

**Authors:** Maria Donata Di Taranto, Asier Benito-Vicente, Carola Giacobbe, Kepa Belloso Uribe, Paolo Rubba, Aitor Etxebarria, Ornella Guardamagna, Marco Gentile, Cesar Martín, Giuliana Fortunato

**Affiliations:** 1 Dipartimento di Medicina Molecolare e Biotecnologie Mediche, Università degli Studi di Napoli Federico II, Napoli and CEINGE S.C.a r.l. Biotecnologie Avanzate, Napoli, Italy; 2 Biofisika Institute (CSIC, UPV/EHU) and Departamento de Bioquímica, Universidad del País Vasco, Apdo. 644, 48080 Bilbao, Spain; 3 Dipartimento di Medicina Clinica e Chirurgia, Università degli Studi di Napoli Federico II, Napoli, Italy; 4 Dipartimento di Scienze della Sanità Pubblica e Pediatriche, Università degli Studi di Torino, Torino, Italy

## Abstract

Familial hypercholesterolemia (FH) is an autosomal dominant disease caused by pathogenic variants in genes encoding for LDL receptor (LDLR), Apolipoprotein B and Proprotein convertase subtilisin/kexin type 9 (PCSK9). Among PCSK9 variants, only Gain-of- Function (GOF) variants lead to FH. Greater attention should be paid to the classification of variants as pathogenic. Two hundred sixty nine patients with a clinical suspect of FH were screened for variants in LDLR and the patients without pathogenic variants were screened for variants in PCSK9 and APOB. Functional characterization of PCSK9 variants was performed by assessment of protein secretion, of LDLR activity in presence of PCSK9 variant proteins as well as of the LDLR affinity of the PCSK9 variants. Among 81 patients without pathogenic variants in LDLR, 7 PCSK9 heterozygotes were found, 4 of whom were carriers of variants whose role in FH pathogenesis is still unknown. Functional characterization revealed that two variants (p.(Ser636Arg) and p.(Arg357Cys)) were GOF variants. In Conclusions, we demonstrated a GOF effect of 2 PCSK9 variants that can be considered as FH-causative variants. The study highlights the important role played by functional characterization in integrating diagnostic procedures when the pathogenicity of new variants has not been previously demonstrated.

## Introduction

Familial Hypercholesterolemia (FH) is a severe genetic hyperlipidaemia characterized by increased levels of LDL cholesterol accumulating in tissues and leading to premature atherosclerosis, tendon xanthomas and corneal arcus. The increased LDL cholesterol levels are due to an altered mechanism of LDL uptake caused by a defect of the involved proteins. The disease is inherited in an autosomal dominant manner and is mainly caused by pathogenic variants in the genes encoding for the LDL receptor (*LDLR*) and its ligand: the Apolipoprotein B (*APOB*)^1^, i.e. the only apolipoprotein of the LDL. The most recently identified FH causative gene encodes for the Proprotein Convertase Subtilisin/Kexin type 9 (*PCSK9*), a secreted protease regulating the LDLR expression post-transcriptionally^2^. The protease PCSK9 is produced as a proprotein that undergoes an autocatalytic cleavage which produces a catalytic domain directly interacting with the cleaved prosegment that functions as an inhibitor. After secretion, PCSK9 binds to LDLR extracellularly and is endocyted with the LDLR-LDL complex through clathrin coated pits. The LDLR bound to PCSK9 is directed toward the lysosome degradation rather than toward the cell membrane recycling. Increased PCSK9 level or function leads to decreased LDLR recycling and to decreased LDLR levels on the cell membrane available for LDL uptake^3^. Another mechanism independent of endocytosis, indicating an intracellular action of PCSK9 in LDLR expression regulation, was observed^4^.

The discovery of PCSK9 as a player of LDL uptake opened new therapeutic avenues. In fact PCSK9 has become the target of several therapies administered in case of failure of traditional therapies or in the most severe cases^5^. Anti-PCSK9 monoclonal antibodies are the most promising^6,7^.

Two different types of pathogenic variants have been identified in this gene: 1. loss of function (LOF) variants producing a less functioning protein, causing an increase of LDLR amounts on the cell membrane and, consequently, hypocholesterolemia; 2. gain of function (GOF) variants producing a more functioning protein that degrades LDLR more efficiently, decreasing its levels and causing FH^8^.

An accurate evaluation of each variant identified during genetic screening is essential to define its pathogenic role. Although several bioinformatics tools are now available^9^, *in silico* predictions are not sufficiently effective to reliably assess the pathogenicity of variants and in particular of GOF variants^10^. Therefore, functional characterization by *in vitro* assays is the most effective and reliable method to evaluate the pathogenic role of gene variants and it is especially required for evaluating PCSK9 variants. Recent guidelines support this concept and suggest that, among different criteria, functional assays can provide strong evidence of pathogenicity^11^. Several methods have been proposed to functionally characterize FH causative variants^12–14^. We report herein the characterization of 4 rare variants in the PCSK9 gene following 3 different approaches.

## Results

### Genetic screening

The screening of *LDLR* gene revealed the presence of variants causative of FH in 188 patients out of 269. In the remaining 81 patients the genetic screening of *PCSK9* revealed the presence of 7 rare missense variants at heterozygous status (2.6% of total patients): 3 variants previously identified in FH patients; 4 variants never associated to FH before. Table 1 reports data about the 7 *PCSK9* variants together with the lipid profile of carriers. The *PCSK9* variant c.1906A > C (p.(Ser636Arg)) was identified in a patient carrying also a very rare variant in the *LDLR* gene, the c.1336 C > G (p.(Leu446Val)). This *LDLR* variant has been found in ExAC with a MAF of 0.0008% and in EVS with a MAF of 0.0077% and was never reported as causative of FH. After performing *in vitro* assays to test the variant effects through the evaluation of the protein expression, the LDL binding and the LDL uptake, we concluded that the *LDLR* variant c.1336 C > G (p.(Leu446Val)) is not causative of FH (Supplementary Fig. S3).

**Table 1:** PCSK9 rare variants identified and characteristics of carrier patients.

Patient ID	Nucleotide substitution	Protein change	Variant ID, MAF* and reference	Total cholesterol (mmol/L)	LDL cholesterol (mmol/L)	HDL cholesterol (mmol/L)	Triglycerides (mmol/L)	Age (years)	Sex
FH-1	c.103 G > T	p.(Asp35Tyr)	rs764603059; gnomAD: 0.00001; NF^†^ ExAC, EVS, 1 kG^16^	8.7	5.8	1.9	2.2	21	Female
FH-2	c.991 C > G	p.(Pro331Ala)	NF^†^ ExAC, gnomAD, EVS, 1 kG	7.7	4.8	2.4		55	Female
FH-3	c.1069 C > T	p.(Arg357Cys)	rs148562777; ExAC: 0.00015; gnomAD: 0.00015; EVS: 0.0002; NF^†^ 1 kG	4.6 ^‡^	2.9^‡^	1.3^**‡**^	0.8^‡^	9	Male
FH-4	c.1394 C > T	p.(Ser465Leu)	rs778849441; gnomAD: 0.00002; NF^†^ ExAC, EVS, 1 kG^17,18^	11.6	8.4	1.5	3.7	65	Female
FH-5	c.1405 C > T	p.(Arg469Trp)	rs141502002; ExAC:0.0007 (0.007 in African; 0.0003 in others); gnomAD: 0.00087 (0.009 in African; 0.0005 in others); EVS: 0.0027 (only African/American); 1 kG: 0.0018 (only African)^19–21^	5.0	3.2	1.5	0.4	8	Female
FH-6^§^	c.1906A > C	p.(Ser636Arg)	NF^†^ ExAC, gnomAD, EVS, 1 kG	9.4	6.6	2.0	1.8	21	Female
FH-7	c.1928A > G	p.(His643Arg)	NF^†^ ExAC, gnomAD, EVS, 1 kG	7.4	5.4	1.4	1.2	30	Male

^*^Minor Allele Frequency according to exome/genome databases (ExAC: Exome Aggregation Consortium; gnomAD: Genome Aggregation Database; EVS: Exome Variant Server; 1 kG: 1000 Genomes).

^†^NF = Not found.

^‡^Biochemical values evaluated during a very restrictive diet.

^§^The patient also carries the LDLR variant p.(Leu446Val) that does not cause alteration of LDLR activity.

The patients bearing PCSK9 rare variants were also screened for APOB variants in order to exclude the presence of additional variants causing FH and no rare variants were found. For the 4 *PCSK9* rare variants never associated to FH, we performed the in silico predictions of pathogenicity (Supplementary Table S1) and we characterize the function of variant protein by several approaches.

### Secretion of p.(Pro331Ala), p.(Arg357Cys), p.(Ser636Arg) and p.(His643Arg) PCSK9 variants to the extracellular medium

**Figure 1:**
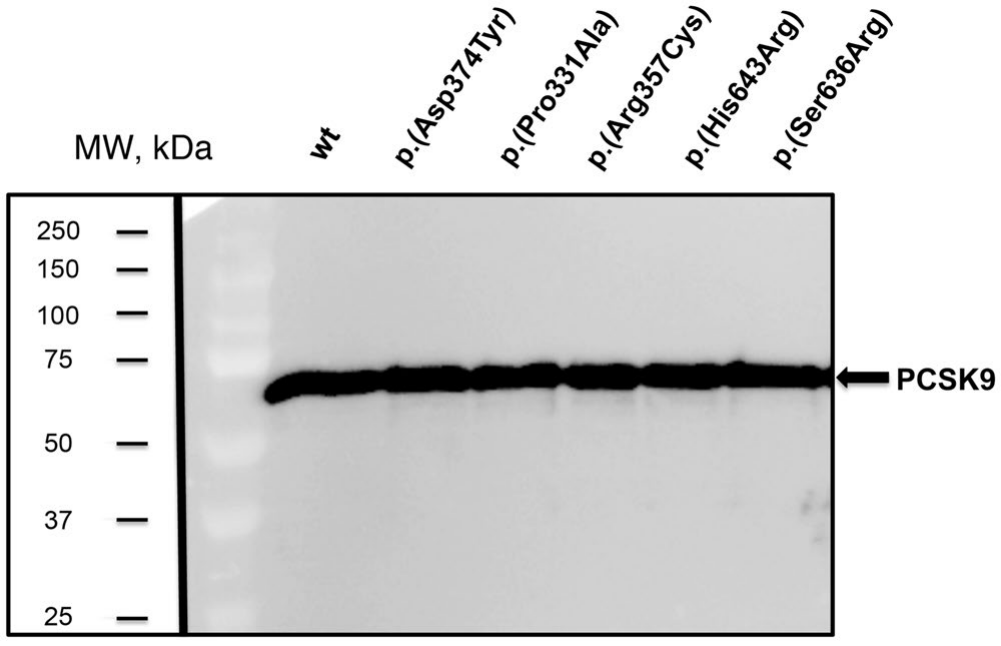
Expression of PCSK9 variants into culture medium of stably transfected HEK293 cells. PCSK9 secretion was analysed by Western blot. The different PCSK9 variants were purified from the culture medium as described in Materials and Methods and 5 µg total protein was subjected to Western blot analysis. A representative experiment from three independently performed assays is shown. The final line of the blot has been cropped because it is a variant not described in this work. The image is a merge of the nitrocellulose membrane showing the pre-stained MW markers and the signal of the western blot. Image bright has been modified in the whole blot. Original membrane and blot can be found in Supplementary Figures S1 and S2 respectively.

HEK293 cells were transfected with DNA constructs encoding for wild-type (wt), p.(Asp374Tyr), p.(Pro331Ala), p.(Arg357Cys), p.(Ser636Arg) and p.(His643Arg) PCSK9 variants and examined the effects of those variants on PCSK9 secretion to the culture medium by Western blot. As shown in Fig. 1, secretion of all the variants was similar than wt PCSK9 secretion. Relative values of PCSK9 determined by ELISA to total cellular protein showed no differences among expression of the different PCSK9 variants compared to wt PCSK9. The secreted protein in the transfected cells expressed as ng PCSK9/total PCSK9 were: wt 16.2 ± 0.8; p.(Asp374Tyr): 15.5 ± 0.1; p.(Pro331Ala): 16.3 ± 0.5; p.(Arg357Cys): 15.7 ± 0.5; p.(Ser636Arg): 15.1 ± 0.9 and p.(His643Arg): 15.7 ± 0.7).

### p.(Arg357Cys) and p.(Ser636Arg) PCSK9 variants diminish LDL uptake activity

**Figure 2:**
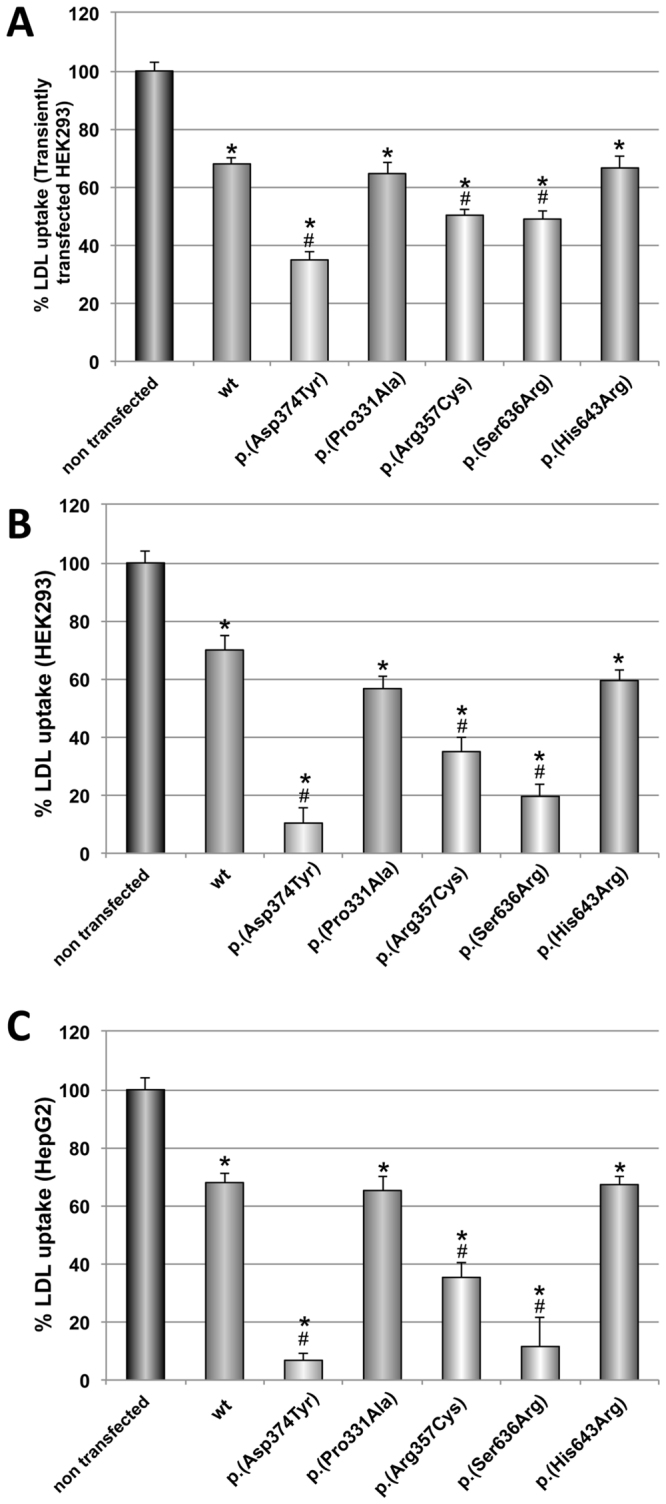
Effect of PCSK9 variants on LDL uptake in (**A**) transiently transfected HEK293 cells, (**B**) in HEK293 cells and (**C**) HepG2 cells incubated with purified PCSK9 variants. LDL uptake in transiently transfected HEK293 cells (**A**) was determined as described in Materials and Methods. For LDL uptake assay with purified PCSK9 variants, cells were incubated with the PCSK9 variants at 0.5 μg/mL during 2 h prior FITC-LDL addition (**B** and **C**). LDL internalization was determined after 4 h incubation at 37 °C as described in Materials and Methods. Values represent the mean ± standard deviation of 3 independent experiments performed by triplicate. *p < 0.01 versus no PCSK9 addition; ^#^p < 0.01 versus wild-type (wt) PCSK9. p.(Asp374Tyr) GOF mutant was used as internal control.

In the first experimental approach to determine the activity of the PCSK9 variants, HEK293 cells were transiently transfected with wt, p.(Pro331Ala), p.(Arg357Cys), p.(Ser636Arg), p.(His643Arg) expression vectors (GOF p.(Asp374Tyr) PCSK9 variant was used as a positive internal control of the method). The efficiency of fluorescent LDL (FITC-LDL) uptake by the cells was measured as described in materials and methods. As shown in Fig. 2A, LDL uptake was significantly reduced upon expression of p.(Arg357Cys) and p.(Ser636Arg) PCSK9 variants (≈25%), compared to wt PCSK9. The p.(Asp374Tyr) variant (used as a GOF control) was the most potent in reducing LDL uptake in those cells (≈50%) while p.(Pro331Ala) and p.(His643Arg) variants showed similar activities than wt PCSK9 (Fig. 2A).

This approach investigates both intracellular and extracellular effects of PCSK9 variants. In order to deeply investigate the action mechanism of PCSK9 variants and to test only their extracellular action, the LDLR assay was performed incubating cells in a culture medium and adding the recombinant purified PCSK9 variants exogenously. For that purpose, HEK293 and HepG2 cells (to test the PCSK9 behaviours in a hepatic cell line), Fig. 2B and C, respectively, were treated with 5 μg/mL PCSK9 variants. Then, cells were incubated with 20 μg/mL FITC-LDL to determine the extent of LDL uptake. As shown in Fig. 2B, p.(Arg357Cys) and p.(Ser636Arg) showed a GOF activity in HEK293 cells in which LDL uptake was diminished significantly when compared to wt PCSK9. Activities of p.(Pro331Ala) and p.(His643Arg) PCSK9 variants were similar to wt PCSK9. The p.(Asp374Tyr) GOF variant was used as internal control of the assay, and caused the expected reduction of LDL uptake already described. Similarly, GOF activities of p.(Arg357Cys) and p.(Ser636Arg) variants, were confirmed by assessing FITC-LDL uptake in HepG2 cells. As shown in Fig. 2C, p.(Arg357Cys) and p.(Ser636Arg) variants showed GOF activities while p.(Pro331Ala) and p.(His643Arg) PCSK9 variants resulted non-pathogenic with similar effects than wt PCSK9.

### p.(Arg357Cys) and p.(Ser636Arg) PCSK9 variants show higher affinity for LDLR than wt PCSK9

**Figure 3:**
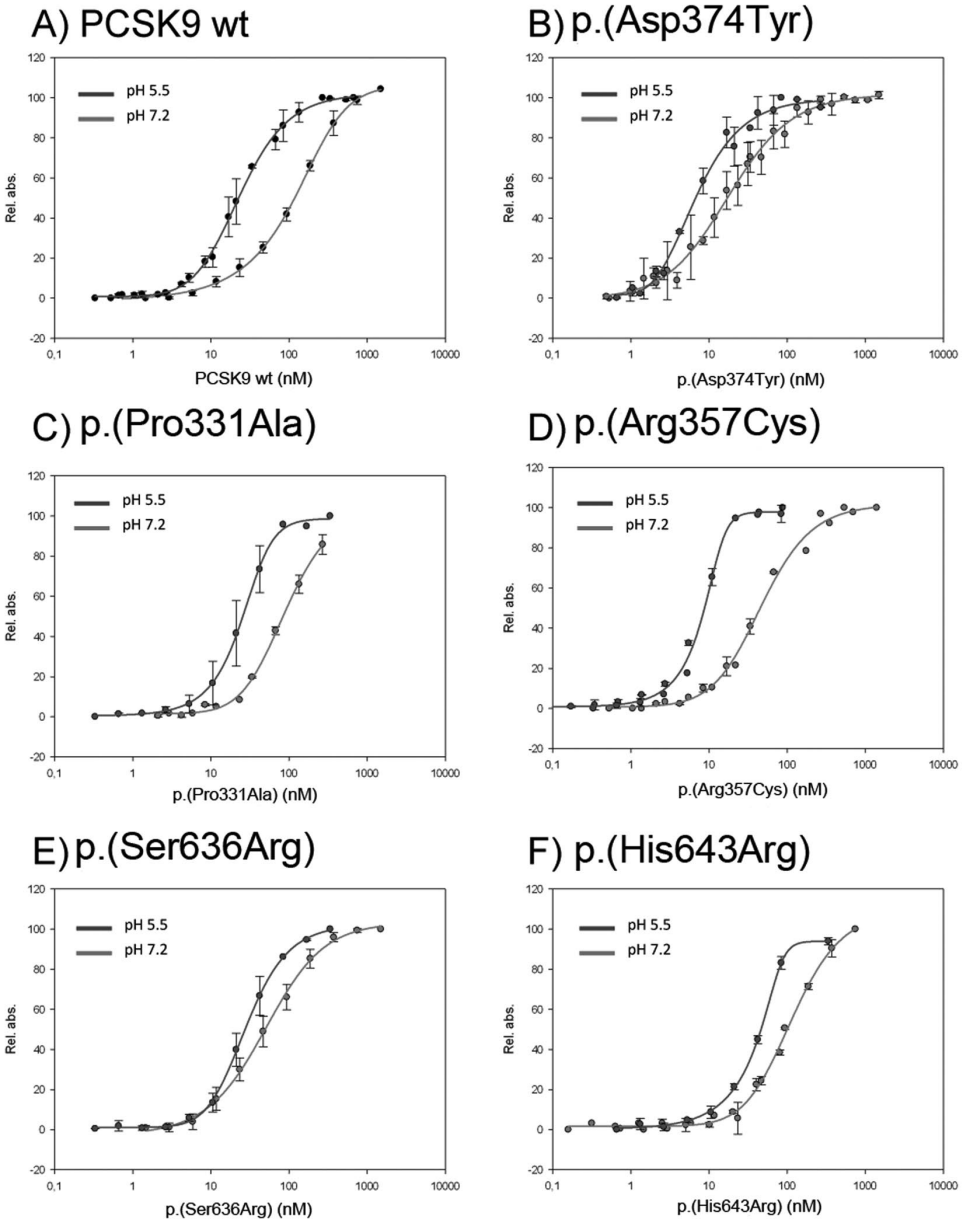
Binding affinity curves of wild-type and PCSK9 variants for LDLR at both pH 7.2 and pH 5.5. Panel (A) wild-type (wt) PCSK9; panel (B) p.(Asp374Tyr); panel (C) p.(Pro331Ala); panel (D) p.(Arg357Cys); panel (E) p.(Ser636Arg) and panel F) p.(His643Arg). Solid-phase immunoassay for PCSK9-LDLR ectodomain binding was performed as described in Methods. Values represent the mean ± standard deviation of 3 independent experiments performed by triplicate.

Next, we tested binding affinities of wt PCSK9, p.(Pro331Ala), p.(Arg357Cys), p.(Ser636Arg) and p.(His643Arg) PCSK9 variants for LDLR at both pH 7.2 and pH 5.5 using a solid phase binding immunoassay. Figure 3 shows the LDLR-PCSK9 binding curves obtained with purified LDLR ectodomain incubated at a fixed concentration with serial dilutions of each PCSK9 variant. The EC_50_ values calculated from these binding curves are shown in Table 2, indicating that at pH 7.2 the EC_50_ for wt LDLR is 112.2 nM, very similar to the previously reported values^15^. Affinity values of p.(Pro331Ala) variant to LDLR were similar to wt PCSK9, whereas for the p.(His643Arg) variant, only the value at pH 7.2 is similar to the wt, being its EC_50_ at pH 5.5 higher than the wt (44.2 nM *vs*. 23.2 nM, respectively) (Table 2).

**Table 2:** EC_50_ values for the binding of PCSK9 variants to LDLR, as determined by solid-phase immunoassay at pH 7.2 and pH 5.5.

	EC_50_ (nM)^*^	
	pH 7.2	pH 5.5
Wild-type	112.2 ± 16.8	23.2 ± 3.7
p.(Asp374Tyr)	19.3 ± 9.4	7.4 ± 1.7
p.(Pro331Ala)	97.6 ± 21.7	25.5 ± 7.7
p.(Arg357Cys)	50.9 ± 13.6	13.3 ± 6.7
p.(Ser636Arg)	47.4 ± 7.1	13.9 ± 5.5
p.(His643Arg)	93.7 ± 13.4	44.2 ± 3.2

^*^Data are reported as mean ± standard deviation.

However, EC_50_ values for wt LDLR of p.(Arg357Cys) and p.(Ser636Arg) variants were 50.9 and 47.4 nM, respectively, thus showing a higher affinity for LDLR and confirming the GOF activity. In addition and as internal control for method validation, EC_50_ value of p.(Asp374Tyr) GOF was determined, and as expected, this variant shows a higher affinity to LDLR compared to wt PCSK9 (19.3 nM *vs*. 112.2 nM, respectively) (Table 2). EC_50_ values for wt LDLR of the different variants was also determined at acid pH (5.5) but no differences among them were found, being the affinities of p.(Arg357Cys) and p.(Ser636Arg) variants very similar to p.(Asp374Tyr) GOF variant, and higher than those of wt, p.(Pro331Ala), and p.(His643Arg) PCSK9 variants (Table 2).

## Discussion

Variants in the PCSK9 gene are responsible for about 1% of FH cases, whereas the LDLR variants account for most of cases^1^. The action mechanism of PCSK9 is at the basis of the dual effect of its variants, LOF variants causing hypocholesterolemia and GOF variants causing FH. The rare variants in the PCSK9 gene should be correctly evaluated before claiming their role as LOF or GOF. According to recent guidelines, a strict pathogenic classification is needed to correctly define the role of variants identified during re-sequencing studies. Functional studies are qualified as “strong” criteria for assessing variant pathogenicity^11^.

In this study, 7 rare variants in the PCSK9 gene (2.6% of total examined population) were identified, 4 of which had never been previously described in FH patients. The prevalence of FH-causing variants in PCSK9 was 1.8% when considering only the GOF variants, i.e. the 3 previously identified variants^16–21^ together with the 2 variants identified in this study. The prevalence of PCSK9 variants causative of FH varies among different countries being very low in the Dutch and English populations (0.1–2%)^22^ and higher in the French population, reaching 5% in the study of Abifadel *et al*.^16^. However, in some studies only the most frequent PCSK9 variant p.(Asp374Tyr) was searched^23^ or only rare variants already annotated as pathogenic are reported^24^, partially justifying the very low frequency of PCSK9 rare variants.

In order to test the effect of PCSK9 rare variants on LDLR, functional characterization was performed by assessing protein secretion, LDLR activity in the presence of PCSK9 variant proteins as well as LDLR affinity of PCSK9 variants. Although the amounts of secreted PCSK9 appeared to be similar among the studied variants and the wt protein, we observed different behaviours of the LDLR regulation: 2 variants were GOF and 2 variants did not show any alteration compared to the wt PCSK9. The effect of PCSK9 variants on LDLR was first tested on HEK293 transiently transfected with each PCSK9 variant, and then on HEK293 and HepG2 incubated with purified PCSK9 variants. These two approaches were used to test both intracellular and extracellular action of PCSK9 variants, showing concordant results. The measurement of LDLR affinity of the 4 variants evaluated in solid phase at both neutral and acid pH confirmed the GOF effect of the p.(Arg357Cys) and p.(Ser636Arg) variants with almost doubled affinity compared to the wt protein.

The p.(Arg357Cys) variant was identified in a 9 years old child, showing a mildly altered lipid profile although it was evaluated while the child was following a very restrictive diet. The variant affected the same amino-acid as the one of p.(Arg357His)^19^ previously identified in a FH patient. Our p.(Arg357Cys) variant could causes the creation of a new potential site for disulphide bond adjacent to the Cys358, normally involved in a disulphide bond near the active site of the protein. Functional data clearly showed the GOF effect of this variant, with a significant increase in PCSK9 activity compared to the wt PCSK9.

The p.(Ser636Arg) variant was identified in a woman with a severe lipid profile also bearing the LDLR p.(Leu446Val) variant. We firstly performed 3 different experiments to demonstrate that this LDLR variant does not decrease LDLR expression and activity. After screening the patient for the other FH-causing genes, the PCSK9 p.(Ser636Arg) variant was found and its GOF effect was demonstrated. Although the p.(Ser636Arg) variant has never been described in the ExAC, EVS and 1000 genomes databases, a functional role of this amino-acid substitution has been hypothesized by Geschwindner *et al*. since the positive charged arginine may cause increased affinity of PCSK9 towards LDLR^20^. The authors *de novo* designed and characterized the same p.(Ser636Arg) variant as the one found in our FH patient revealing a mild LOF effect. The differences between our results and those previously reported^20^ could be explained by a possible different pattern of post-translational modification due to the production of proteins in insect cells instead of human cells. In our results, this variant shows a highly increased extracellular activity of PCSK9, similar to the severe GOF p.(Asp374Tyr) variant, used as a positive control. This is in agreement with the severe lipid profile observed in our FH patient.

Furthermore, we demonstrated that the PCSK9 p.(Pro331Ala) and p.(His643Arg) variants do not cause any increased protein function and cannot be considered as being causative of FH. In the case of p.(His643Arg), affinity values of wt and p.(His643Arg) variant, are similar at pH 7.2, whereas its EC_50_ at pH 5.5 is higher than wt (44.2 nM *vs*. 23.2 nM, respectively). This weaker affinity of p.(His643Arg) variant toward LDLR at low pH could indicate an increased dissociation rate in late endosomes. However, this lower affinity upon acidification does not lead to a LOF effect as shown by a similar LDL uptake in HEK293 and HepG2 cells treated with wt or p.(His643Arg) PCSK9 variant. The *in vitro* results obtained with p.(His643Arg) are also confirmed by the LDL-cholesterol levels of patient FH-7.

As for the 3 variants previously described in other studies on FH patients, the p.(Asp35Tyr) variant was identified in a French patient by Abifadel *et al*. who reported the variant as being responsible for a novel Tyr-sulfation site creation, which may enhance the intracellular activity of PCSK9^16^. The p.(Ser465Leu)was previously reported by our group as a variant associated with an extremely variable FH phenotype^17^. A subsequent study reported the presence of this variant in 10 patients from the Netherlands with a mild hypercholesterolemia^18^. The variant p.(Arg469Trp) was firstly identified in a double heterozygote patient also carrying a null variant in the LDLR gene^19^. The variant was also described by Kotowski *et al*. in a multi-ethnic population from the United States: black carrier subjects showed a variable lipid profile ranging from low to high LDL cholesterol, whereas the only white carrier showed increased LDL levels^21^. In the next generation sequencing databases ExAC and gnomAD, the variant p.(Arg469Trp) was reported at higher frequency in African populations respect to other populations, whereas in the EVS and 1 kG the variant was exclusively found in African derived population. The presence of other in linkage variants might be responsible for the different phenotypes observed in the two ethnic groups. The functional characterization performed by Geschwindner *et al*.^20^ with the PCSK9 variant purified from insect cells and incubated on HepG2 cells revealed a very mild LOF effect, although this approach might be influenced by the use of a non-human protein-production system.

In conclusion, in our study 4 PCSK9 variants with an unknown effect on FH pathogenesis were identified. After extensive functional evaluation, we demonstrated a GOF effect of the p.(Arg357Cys) and p.(Ser636Arg) variants that could be considered as FH causative variants.

## Methods

### Patients and genetic screening

Based on biochemical and clinical features, 269 patients were suspected of FH and enrolled in this study. Simon Broome criteria were followed for patient inclusion with the exception of paediatric patients that were also included if LDL cholesterol levels were higher than 90^th^ percentile^25^ and a clear hypercholesterolemia was found in a parent.

The study was performed according to the current version of the Helsinki Declaration and was approved by the Ethical Committee of the Università degli Studi di Napoli Federico II (Number 157/13, September 9, 2013). Informed consent was obtained for each patient. All patients underwent the genetic screening of the *LDLR* gene by amplification of promoter, exons and exon-intron junctions followed by direct sequencing as previously described^26^. If no pathogenic variants were detected, Multiplex Ligation-dependent Probe Amplification (MLPA) was performed as previously reported^26^ to search for large rearrangements in the *LDLR* gene. Molecular analysis of the *PCSK9* gene was performed in patients without pathogenic variants in the *LDLR* gene. *PCSK9* screening included the amplification and direct sequencing of promoter, exons and exon-intron junctions^27^. APOB screening included the analysis of the region coding for the LDLR binding region, i.e. a portion of the exon 26 (c.9670-c.11916, p.Lys3181-p.Asn3929) and the exon 29 ^27^.

Identified variants were checked against pathogenic variants databases: Leiden Open Variation Database (LOVD) and Human Gene Mutation Database (HGMD). Variants not present in these databases or never reported as causative of FH in literature were searched in next generation sequencing databases: Exome Aggregation Consortium (ExAC), Genome Aggregation Database (gnomAD - it consists in ExAC data integrated with additional exome and genome data); Exome Variant Server (EVS) and 1000 genomes. Rare variants are defined as variants with a Minor Allele Frequency (MAF) less than 1%.

Bioinformatics predictions included PolyPhen-2 (Polymorphism Phenotyping v2), MutationTaster, PMUT, Sorting Intolerant from Tolerant (SIFT) and Protein Variation Effect Analyzer (PROVEAN).

### LDLR-ectodomain production and purification

The LDLR construct encoding the N-terminal extracellular ectodomain (1–789 amino acids) plus c-myc and His tags was purified by affinity chromatography from cells transfected with the pcDNA3.1-EC-*LDLR*-His plasmid, kindly provided by Prof. Leren^28^. Briefly, HEK293 cells at 70–80% confluency were transfected with the plasmid by calcium phosphate method for 24–48 h and selected in successive passages by geneticin (G-418 sulphate, Gibco, Invitrogen). For EC-LDLR expression and purification, the growing medium of positively transfected cells was changed to Opti-MEM (Invitrogen) without geneticin and maintained under these conditions for other three days. Then the medium was harvested, supplemented with protease inhibitors (complete EDTA-free, Roche) and the LDLR ectodomain was affinity purified using one-step nickel affinity chromatography. For protein long-term maintenance, the buffer was changed to storage buffer (50 mM Tris-HCl, 50 mM NaCl, 10% glycerol, and 0.01% Brij-35, pH 7.5)^29^ and frozen to −80 °C.

### Site-directed mutagenesis and cloning

Plasmids carrying *PCSK9* variants were constructed by Innoprot (Derio, Spain). Briefly, variants were introduced into the human PCSK9 cDNA (NM_174936.3), in the mammalian expression vector wt-PCSK9 plasmid (pCMV-PCSK9-FLAG) kindly provided by Prof. Horton^30^. Variants were introduced by oligonucleotide site-directed mutagenesis using QuickChange Lightning mutagenesis kit (Agilent) according to the manufacturer’s instructions. A 6x His tag was introduced to allow purification. Restriction enzyme digestion of the appropriate fragments and the integrity of the remaining PCSK9 cDNA sequence of all constructs were verified by direct sequence analysis.

### PCSK9 expression in transfected HEK293 cells and Western blot analyses

HEK293 cells at a confluence of 5 × 10^5^ cells/well in 6-well culture plates (Sarstedt, Germany) were transfected with 1 µg cDNAs with Lipofectamine® LTX and PlusTM Reagent (Invitrogen). 24 h post-transfection, cells were washed and then incubated with fresh DMEM medium for an additional 24 h and then, media was recovered and secretion of PCSK9 was analysed by Western blot. For that purpose, proteins in the media were resolved by 8.5% Tris-Glycine SDS-PAGE. The gels were blotted onto Nitrocellulose membranes (Protran BA 83, Whatman™, GE Healthcare, Germany), blocked for 1 h in TBS-T (50 mM Tris-HCl, pH 7.5, 150 mM NaCl, 0.1% Tween 20) containing 5% non-fat milk and immunoblotted with a rabbit polyclonal anti-human PCSK9 antibody (1:1000) (Cayman Chemical Company, USA, Cat.No: 10240) for 16 h at 4 °C. Then, counterstained with a horseradish peroxidase-conjugated anti-rabbit antibody (Cell Signalling, Cat.No: 7074 s). The signal was developed using SuperSignal West Dura Extended Substrate (Pierce Biotechnology, Rockford, IL, USA).

### PCSK9 purification from stably transfected HEK293 cells

HEK293 cells grown to subconfluence were transfected with the different PCSK9 plasmids and subcultured with geneticin G418 sulphate (Gibco) according to the manufacturer’s instructions to obtain the stably transfected cells. For PCSK9 purification, stably transfected HEK293 cells were grown at 80% confluence in DMEM medium, then, culture medium was replaced by Opti-MEM (Invitrogen) without geneticin and cells were maintained under these conditions for 48 h. Finally, the medium was harvested and PCSK9 was purified using one-step nickel affinity chromatography. Purified PCSK9 variants were stored at −80 °C in 50 mM Tris-HCl buffer supplemented with 150 mM NaCl and 10% glycerol, pH 8.0.

### Lipoprotein labelling with FITC

LDL was labelled with FITC as previously described^31^. Briefly, 10 µL of FITC (2 mg/mL) were added to 1 mL LDL (1 mg/mL) in 0.1 M NaHCO_3_, pH 9.0, was mixed for 2 h by slow rocking at room temperature. The unreacted dye was removed by gel filtration on a sephadex G-25 column equilibrated with PBS EDTA-free buffer. All fractions were assayed for protein content using bovine serum albumin as standard (Pierce BCA protein assay, Pierce).

### Quantification of LDL uptake by flow cytometry

48 h after transfection with the plasmids containing the different PCSK9 variants, HEK293 cells were incubated for 4 h, at 37 °C with 20 µg/mL FITC-LDL and lipoprotein uptake was determined as previously described^31^. In addition, LDL uptake was determined using purified PCSK9 variants in HepG2 cells and HEK293 cells. Briefly, 2 µg/mL of each purified PCSK9 variant was added to the cell culture medium and 2 h post-addition, 20 µg/mL FITC-LDL was added to the medium and LDL uptake was determined 4 h after lipoprotein addition. In both experimental approaches, after incubation with FITC-LDL, cells were washed twice in PBS-1%BSA, fixed on 4% formaldehyde for 10 min and washed again twice with PBS-1%BSA. The amount of internalized LDL was determined as described before by adding Trypan blue solution (Sigma-Aldrich, Steinheim, Germany) to a final concentration of 0.2% (Etxebarria *et al*., 2014). Fluorescence intensities were measured in a FACSCalibur™ (BD Bioscience, NJ, USA) flow cytometer as previously described^31^. For each sample, fluorescence of 10,000 events was acquired for data analysis. All measurements have been performed at least in triplicate.

### Solid-phase immunoassay for PCSK9-LDLR ectodomain binding

Purified LDLR ectodomain diluted in working buffer (10 mM Tris-HCl, pH 7.5, 50 mM NaCl, 2 mM CaCl_2_) was coated at a fixed concentration onto 96-well microtiter plates by incubation ON at 4 °C. Plates were then blocked and incubated with a serial dilution of each sample diluted in working buffer during 2 hours at RT, and then washed thoroughly with working buffer supplemented with 0.1% (w/v) Tween 20 (Sigma-Aldrich, MO, USA). For ligand detection, the antibodies (mouse monoclonal anti-DDK, clone OTI4C5, Origene, USA; and peroxidase-conjugated horse anti-mouse, Cell Signaling, USA) were diluted in working buffer supplemented with 5% (w/v) BSA, applied directly to the plate and incubated for 1 hour at RT, with an extensive washing between both incubations. After a final wash, antibody binding was determined using 50 μL per well of 2,2´-Azino-bis (3-ethylbenzothiazoline-6-sulfonic acid) substrate solution (Sigma-Aldrich, MO, USA) and measuring colour change at 405 nm. The time course for colour development was essentially linear and measurements were taken 30–60 min after the addition of substrate. For data processing, all absorbance values were corrected for unspecific binding, relativized to maximum and EC_50_ values were extracted from curves after fitting the data to 5-parameter logistic (5-PL) equation (SigmaPlot 13.0, Systat Software Inc., CA, USA).

## Electronic supplementary material

Supplementary data
