# Supplementary material for: Identification and in vitro characterization of two new PCSK9 Gain of Function variants found in patients with Familial Hypercholesterolemia
Source: Sci Rep. 2017 Nov 10;7:15282. doi: 10.1038/s41598-017-15543-x (PMC5681505; doi:10.1038/s41598-017-15543-x)
Supplement: Supplementary file 1 — Supplementary data [file 41598_2017_15543_MOESM1_ESM.doc]

**SUPPLEMENTARY DATA**

Research article

**Identification and *in vitro* characterization of two new PCSK9 Gain of Function variants found in patients with Familial Hypercholesterolemia**

Maria Donata Di Taranto^1#^, Asier Benito-Vicente^2#^, Carola Giacobbe^1^, Kepa B. Uribe^2^, Paolo Rubba^3^, Aitor Etxebarria^2^, Ornella Guardamagna^4^, Marco Gentile^3^, Cesar Martin^2^* and Giuliana Fortunato^1^*.

**Author affiliations**

^1^ Dipartimento di Medicina Molecolare e Biotecnologie Mediche, Università degli Studi di Napoli Federico II, Napoli and CEINGE S.C.a r.l. Biotecnologie Avanzate, Napoli, Italy

^2^ Biofisika Institute (CSIC, UPV/EHU) and Departamento de Bioquímica, Universidad del País Vasco, Apdo. 644, 48080 Bilbao, Spain

^3^ Dipartimento di Medicina Clinica e Chirurgia, Università degli Studi di Napoli Federico II, Napoli, Italy

^4^ Dipartimento di Scienze della Sanità Pubblica e Pediatriche, Università degli Studi di Torino, Torino, Italy

^#^ these authors contributed equally to the paper

* corresponding authors:

- Giuliana Fortunato, Dipartimento di Medicina Molecolare e Biotecnologie Mediche, Università degli Studi di Napoli Federico II, via S. Pansini 5, 80131 Napoli, and CEINGE Biotecnologie Avanzate s.c. a r.l., via Gaetano Salvatore 486, 80145 Napoli, Italy. Telephone: +39-081.746.4200; email: fortunat@unina.it

- Cesar Martin, Biofisika Institute (CSIC, UPV/EHU) and Departamento de Bioquímica, Universidad del País Vasco, Apdo. 644, 48080 Bilbao, Spain. Telephone: +34-94.601.8053; email: cesar.martin@ehu.eus

**Supplementary Methods**

**Site-directed mutagenesis**

Plasmid carrying the *LDLR* p.(Leu446Val) variant was constructed by Innoprot (Derio, Spain). Briefly, variants were introduced into the human *LDLR* cDNA (NM_000527.4), using the mammalian expression vector pcDNA3 under control of a SV40 promoter by oligonucleotide site-directed mutagenesis using the QuickChange Lightning mutagenesis kit (Agilent) according to the manufacturer's instructions. The oligonucleotides used to generate the plasmid carrying the p.(Leu446Val) LDLR variant in study were synthesized *in vitro* and subcloned using the restriction enzymes SacII and EcoRI. The presence of the desired nucleotide alteration was confirmed by PCR and restriction enzyme digestion of the appropriate fragments. The integrity of the remaining *LDLR* cDNA sequence of all constructs was verified by direct sequence analysis.

**Cell culture and transfection**

*LDLR*-deficient CHO cell line *ldl*A7 (CHO-*ldl*A7) (kindly provided by Dr. Monty Krieger, Massachusetts Institute of Technology, Cambridge, MA) was cultured in Ham’s F-12 medium supplemented with 5% FBS, 2 mM L-glutamine, 100 units/mL penicillin, and 100 μg/mL streptomycin. CHO-*ldl*A7 cells were plated into 6- or 24-well culture plates, and transfected with plasmids carrying the *LDLR* variants using Lipofectamine® LTX and Plus^TM^ Reagent (Invitrogen) according to the manufacturer’s instructions. Transfected cells were maintained in culture for 48 h to achieve maximal LDLR expression.

**Quantification of LDLR activity by flow cytometry**

Transfected CHO-*ldl*A7 cells were grown in 24-well culture plates. 48 h after transfection, cells were incubated for 4 h, at 37ºC or at 4ºC with 20 µg/mL FITC-LDL to determine LDLR activity or LDL-LDLR binding, respectively. After incubation with FITC-LDL, CHO-*ldl*A7 cells were washed twice in PBS-1%BSA, fixed on 4% formaldehyde for 10 min and washed again twice with PBS-1%BSA. To determine the amount of internalized LDL, Trypan blue solution (Sigma-Aldrich, Steinheim, Germany) was added directly to the samples to a final concentration of 0.2%, eliminating the extracellular signal due to the non-internalized LDL-LDLR complexes. Fluorescence intensities were measured by FACS, in a Facscalibur Flow cytometer according to the manufacturer instructions as previously described[^1^](#_ENREF_1). For each sample, fluorescence of 10,000 events was acquired for data analysis. All measurements were performed at least in triplicate.

**Quantification of LDLR expression by flow cytometry**

To determine LDLR cell surface expression by FACS, transfected CHO-*ldl*A7 cells grown for 48 h were incubated with a mouse primary antibody anti-LDLR (1:100; 2.5 mg/L; Progen Biotechnik GmbH) for 1 h, at room temperature, then washed twice with PBS-1%BSA and incubated with secondary antibody Alexa Fluor 488-conjugated goat anti-mouse IgG (1:100; Molecular Probes). For each sample, fluorescence of 10,000 events was acquired for data analysis. All measurements were performed at least in triplicate.

**Supplementary reference**

1. Etxebarria A, Palacios L, Stef M, Tejedor D, Uribe KB, Oleaga A, Irigoyen L, Torres B, Ostolaza H, Martin C. Functional characterization of splicing and ligand-binding domain variants in the ldl receptor*. Human mutati*on. 2012;33:232-243


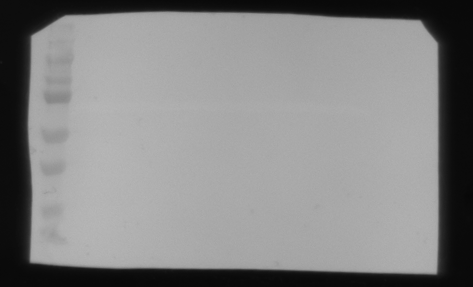


**Supplementary Figure S1: Nitrocellulose membrane from the Western blot shown in Figure 1.** The first lane shows the molecular weight markers. The image is not processed.


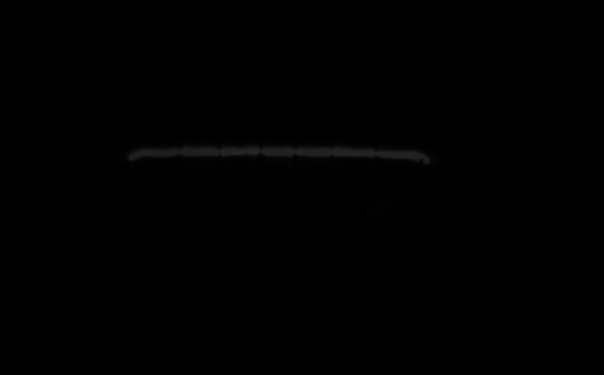


**Supplementary Figure S2: Signal of the Western blot shown in Figure 1.** The figure shows all the bands, including the last one that does not appear in Figure 1 because that variant is not related to this work. The image is not processed.

**Supplementary Figure S3: Functional characterization of p.(Leu446Val) LDLR variants.** **A**: LDLR expression at cellular membrane; **B**: LDL-LDLR binding after 4 h incubation at 4 ºC; **C**: LDL internalization after 4 h incubation at 37ºC. 10,000 cells were acquired in a Facscalibur and values of LDL uptake, binding and LDLR expression were calculated as described in Methods. The values represent the mean of triplicate determinations (n = 3); error bars represent ±SD. *P < 0.01 compared to the wt using a Student’s t-test. p.Trp87* and Ex3_4del LDLR variants were used as internal controls of the method

**Supplementary Table S1. In silico predictions of PCSK9 rare variants never reported as causative of FH**

| **Nucleotide substitution** | **Protein change** | **Polyphen-2 prediction and scores** | **Mutation Taster prediction and reliability probability** | **PMUT prediction and score** | **SIFT prediction and score** | **PROVEAN prediction and score** |
| --- | --- | --- | --- | --- | --- | --- |
| c.991C>G | p.(Pro331Ala) | HumDiv: Probably Damaging 0.948 HumVar: Benign 0.436 | Disease Causing, probability 1 | Neutral, 0.31 | Tolerated, 0.42 | Neutral, -2.25 |
| c.1069C>T | p.(Arg357Cys) | HumDiv: Probably Damaging 1.000 HumVar: Probably Damaging 0.969 | Disease Causing, probability 0.9998 | Disease, 0.59 | Damaging, 0.01 | Deleterious, -3.60 |
| c.1906A>C | p.(Ser636Arg) | HumDiv: Probably Damaging 0.779 HumVar: Benign 0.168 | Polymorphism, probability 0.980 | Neutral, 0.22 | Damaging, 0.02 | Neutral, -1.11 |
| c.1928A>G | p.(His643Arg) | HumDiv: Benign 0.001 HumVar: Benign 0.001 | Polymorphism, probability 0.999 | Neutral, 0.07 | Tolerated, 0.52 | Neutral, -0.71 |
